# Role of Uropathogenic *Escherichia coli* and Other Pathogens in Kidney Stone Formation: From Pathogenesis to Treatment

**DOI:** 10.3390/pathogens14100991

**Published:** 2025-10-01

**Authors:** Beata Zalewska-Piątek, Michalina Nagórka, Rafał Piątek

**Affiliations:** Department of Biotechnology and Microbiology, Chemical Faculty, Gdańsk University of Technology, Narutowicza 11/12, 80-233 Gdańsk, Poland; micnagor@pg.edu.pl (M.N.); rafpiate@pg.edu.pl (R.P.)

**Keywords:** uropathogenic *E. coli*, urinary tract infections, kidney stones, nephrolithiasis, urolithiasis, calcium oxalate, nucleation, lithogenic mechanism, therapeutic interventions

## Abstract

Urinary tract infections (UTIs) are among the most prevalent infections in the human population. Uropathogenic *Escherichia coli*, the primary causative agent of UTIs, may also contribute to the development of metabolic kidney stones, particularly those composed of calcium oxalate. Kidney stone disease (KSD), known as nephrolithiasis or urolithiasis, is one of the most common disorders of the urinary system. This review explores the significant clinical association between UTIs and kidney stones, focusing on the mechanisms by which UPEC may promote stone formation, including oxidative stress, inflammation, and altered citrate metabolism. It also examines the role of immune responses, particularly macrophage activity, in the progression of KSD. Recent evidence suggests that the composition of the gut microbiota and metabolic imbalances have an additional impact on stone development. In light of these findings, current prevention and treatment strategies, including microbiota-targeted therapies, probiotics, and immune modulation, are also reviewed. Understanding the complex links between UTI, immunity, and metabolism provide new insights into the pathogenesis of KSD and allows for the development of more effective treatments for this disease.

## 1. Introduction

Urinary tract infections (UTIs) are one of the most common bacterial infections, affecting 150 million people worldwide each year [1,2]. Evidence links UTIs to kidney stone (KS) formation, one of the most common urinary disorders, also known as nephrolithiasis or urolithiasis [3,4]. Kidney stone disease (KSD) can also be considered a systemic disease influenced by a number of interrelated factors such as gender, environmental conditions, hypertension, diabetes, dietary habits (obesity, for example, can alter urine pH and the composition of excreted substances), and genetic predisposition [4,5,6]. Stones may remain localized in the kidney or migrate through the urinary tract into the ureter. When stones remain anywhere in the urinary tract, they can obstruct the normal flow of urine, potentially causing urine to accumulate in the kidney, ureter, bladder, or urethra, which can result in renal colic [4]. Stones associated with UTIs are and prone to recurrence [7,8].

The global prevalence of KSD is estimated at 2–15% (e.g., 1–5% in Asia, 5–9% in Europe, and 13% in North America) [6,9]. In addition, it is emphasized that urolithiasis affects approximately 5–10% of the population in Western countries, with at least one episode occurring during an individual’s lifetime [10,11]. There has been a significant increase in the incidence of this urological disease over the past 30 years [7]. Although KSD has traditionally been more prevalent in men than in women, recent trends indicate a shift, with increasing incidence in women and a relative decline among men [12,13,14].

KSD remains a global health and economic concern, with an estimated 106 million new cases reported in 2021 about 67% of which affected men [15,16]. While the total number of KSD cases has increased by 26.7% since 2000, the global age-standardized incidence rate has declined by 17.5%, reflecting public health successes. Regionally, prevalence varies considerably, with the highest age-standardized incidence rates observed in Eastern Europe (up to 3560 per 100,000), while the lowest rates were reported in western sub-Saharan Africa (around 606 per 100,000). Although pediatric urolithiasis accounts for only 2–3% of all KS cases, its incidence is rising, especially in Brazil, Russia, India, China, and South Africa. India and Russia exhibit the highest pediatric burden, with peak case estimates of 16,331 and 2442, respectively, while South Africa, with only 532 cases at its peak, shows ongoing gaps in early diagnosis and treatment [4,17].

**Figure 1:**
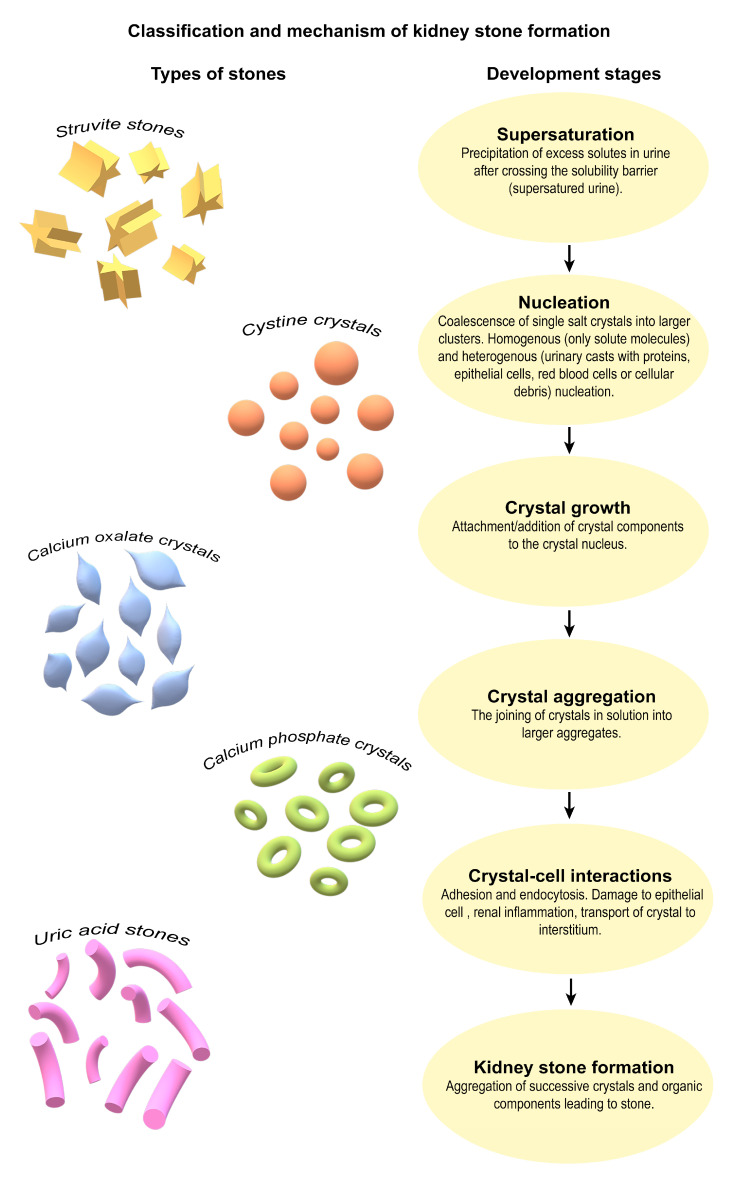
Classification and shared pathophysiological steps in kidney stone formation. The diversity of KS types and the common biological pathway that underlies their formation. On the left, representative morphologies of the five major stone types (struvite, cystine, calcium oxalate, calcium phosphate, and uric acid) are shown to emphasize their compositional heterogeneity. On the right, a unified sequence of physicochemical and cellular events is presented, beginning with urine supersaturation and culminating in stone formation. This schematic highlights that, despite differences in stone composition, the mechanistic steps of crystal initiation, growth, aggregation, and epithelial interaction are shared across stone types. Understanding this convergence may be key to developing broad-spectrum preventive strategies [3,5,8,18,19,20,21,22,23].

KSs are typically composed of inorganic crystalline material (such as calcium oxalate or phosphate), often embedded within an organic matrix consisting of proteins, lipids, and cellular debris. These types of mineral deposits form in the calyces or renal pelvis, either freely or attached to the renal papillae [8]. Calcium oxalate monohydrate (CaOx) stones constituted approximately 65.9% of the KSs analyzed, followed by carbapatite stones (15.6%), urate stones (12.4%), struvite stones (magnesium-ammonium phosphate; 2.7%), and brushite stones (1.7%), reflecting their relative prevalence in the patient population [3,18]. Considering the composition of the stones, it is also possible to distinguish between infectious stones (i.e., calcium stones, brushite, and urate) and non-infectious stones (i.e., carbapatite and struvite) [8]. In general, KSs can be divided into calcareous stones (calcium-containing), accounting for almost 75% of cases, and non-calcareous stones [5]. Among calcareous stones, approximately 50% are primarily composed of CaOx and about 5% consist mainly of calcium phosphate (CaP), occurring separately or in combination (45%) [5,19,20,21]. Oxalate stones are a particular problem and can constitute up to 75–85% of all urinary stones. Tubular deposition of CaOx crystals associated with the formation of KSs is often detected during biopsies of native or transplanted kidneys. In the absence of calcium deposition in the tubules and its deposition in the renal parenchyma, nephrocalcinosis develops [8]. A special type of KSs are cystine stones, which are rare in both children and adults. They arise as a result of inactivating mutations (most commonly substitution of threonine for methionine 467) in genes (e.g., in humans, the *SLC3A1* gene on chromosome 2) encoding renal tubule transporters that reabsorb the amino acid cysteine. Conditions lead to elevated urinary cysteine concentrations in the urine and subsequent stone formation (Figure 1) [22,23].

Classification of stone-forming agents based on stone composition and clinical presentation may facilitate the determination of the risk of future symptomatic stone episodes and assist in tailoring personalized strategies for stone prevention strategies [8,24]. KSs can be highly variable and range from asymptomatic, incidentally detected stones of marginal clinical significance to painful and recurrent stones. Therefore, a potential classification system for KSs should focus mainly on categorizing stone composition and also differentiating symptomatic from asymptomatic stones, confirmed from suspected symptomatic stone episodes. Asymptomatic stones are clinically diagnosed from patient-reported symptomatic stone episodes, and symptomatic stones are diagnosed from radiographic recurrence (such as stone growth, stone disappearance or the appearance of a new stone) [24].

Epidemiological studies have shown a rising incidence of nephrolithiasis in recent years, with recurrence rates reaching up to 50% within five years of the initial stone event. Despite extensive research over the decades, effective treatment options to prevent recurrence are still rare [16,25,26]. Therefore, a deeper understanding of the various aspects of KS formation is required to develop more innovative preventive and therapeutic strategies against KSD.

## 2. Insight into the Mechanisms of Urolithiasis Development

The formation of KSs is fundamentally a physicochemical phenomenon initiated by the supersaturation of urine with stone-forming components such as calcium, oxalate, phosphate and uric acid. When the concentration of these solutes exceeds their solubility, crystallization becomes thermodynamically favorable. This process proceeds through a well-characterized sequence of nucleation, crystal growth, aggregation, and retention. However, it is not a purely passive process. Molecular regulators, including crystallization promoters such as calcium, urate, and oxalate, and inhibitors such as citrate, magnesium, osteopontin, and nephrocalcin modulate the kinetics of these events. Shifting the balance toward promoters fosters crystal formation and stability, while depletion or dysfunction of inhibitors predisposes individuals to stone formation [3,8].

One of the earliest structural factors contributing to the pathogenesis of urolithiasis is the formation of Randall’s plaques of interstitial calcium phosphate deposits located beneath the urothelium of renal papillae [27]. These plaques form in the basement membrane of the thin limbs of Henle’s loop and extend into the interstitium and eventually into the urinary space. Their mineral core serves as a nidus on which CaOx crystals can anchor and overgrow. The plaques consist of a complex matrix of calcium phosphate, collagen, lipids, and vesicular structures, and are increasingly recognized as essential precursors of idiopathic calcium stone disease. Emerging evidence highlights the involvement of osteogenic transformation of interstitial fibroblasts and the regulatory role of long non-coding RNAs in the development of atherosclerotic plaques, pointing to an active, cell-mediated process rather than passive mineral deposition [27,28,29].

At the cellular level, the interaction between renal tubular epithelial cells and crystals is a key step in stone retention and growth. Adhesion of crystals triggers a cascade of proinflammatory responses characterized by oxidative stress, cytokine production and cellular damage. These events further disrupt tubular integrity, facilitating additional crystal binding and propagation [30]. Macrophages, key players in renal immunity, infiltrate these sites and exhibit functional polarization. The dynamic interaction between these macrophage phenotypes, regulated by pathways such as NLRP3 inflammasome and NF-κB, substantially influences stone pathogenesis [30,31].

Sex hormones are also involved in modifying stone risk, contributing to the observed male predominance in CaOx nephrolithiasis. Androgens enhance oxalate biosynthesis and promote tubular epithelial damage and crystal adhesion through several molecular pathways, including activation of glycolate oxidase and NADPH oxidase systems. In contrast, estrogens appear to exert protective effects by downregulating oxidative pathways and reducing the expression of crystal-binding receptors. The differential effects of sex hormones suggest the influence of hormones on both systemic metabolism and the local renal microenvironments, offering potential targets for therapeutic intervention [32,33].

The role of the microbiome in KSD has gained increasing attention, especially after the recognition of both pathogenic and protective effects of microorganisms. Recent advances in molecular techniques, including 16S rRNA gene sequencing and expanded quantitative urine culture (EQUC), have demonstrated that urine is not sterile and that the urinary tract harbors a diverse microbiome, challenging the traditional paradigm [34,35]. Distinct bacterial communities have been identified in different regions of the urinary tract, including the bladder (e.g., *Lactobacillus* spp. and *Gardnerella vaginalis*), urethra (e.g., *Corynebacterium* spp. and *Streptococcus* spp.), and renal pelvis (e.g., *E. coli* and *Enterococcus* spp.), through both molecular and enhanced culture methods [34,35,36,37]. Many of these microorganisms act as commensals that help maintain urinary tract homeostasis, while pathogenic species such as UPEC can disrupt this balance, leading to infection and potentially contributing to kidney stone formation [35,36,37]. Understanding the composition and dynamics of the urinary microbiome is essential for elucidating its role in urolithiasis and other urinary tract diseases [35,37].

Urease-producing bacteria, such as *Proteus mirabilis* and *Klebsiella pneumoniae*, catalyze the hydrolysis of urea to ammonia, increasing urinary pH and promoting the precipitation of magnesium-ammonium phosphate (struvite) stones. Meanwhile, *Calcifying nanoparticles*, ultra-small, calcium-binding particles are hypothesized to promote calcification through oxidative and apoptotic pathways. Although their exact biological nature remains debated, they have been detected in various types of renal stones and are believed to promote calcification through oxidative stress and apoptotic pathways [38]. On the other hand, oxalate-degrading bacteria in the gut, particularly *Oxalobacter formigenes*, reduce oxalate absorption and excretion in the urine, acting as a natural defense against CaOx stone formation [39]. Dysbiosis, including the depletion of such commensal species, has been linked to an increased risk of stone formation, highlighting the systemic dimension of stone pathogenesis [40].

Environmental and systemic factors provide the broader context in which KSs develop. Obesity, insulin resistance, and metabolic syndrome are consistently associated with alterations in urine composition, such as increased acidity, decreased citrate, and increased calcium or oxalate excretion. Diets high in sodium, animal protein, and fructose, combined with inadequate hydration, further increase the risk of urine supersaturation. Additionally, vitamin D deficiency, hyperparathyroidism, and hypomagnesuria disrupt mineral homeostasis and acid-base balance, promoting crystal formation. Genetic predispositions amplified by epigenetic modifications affecting the expression of calcium-sensing receptors and vitamin D metabolism add a hereditary component to the risk profile, affecting susceptibility from generation to generation [5,41].

## 3. Immune Mechanisms Contributing to Calcium Oxalate Crystallization and Kidney Stone Formation

KSD, particularly the formation of CaOx crystals, is a complex process in which the immune system plays a crucial role. In response to the presence of crystals in the kidneys, macrophages are recruited and accumulate at the site, serving as key regulators of both inflammation and stone development [3,30]. These phagocytic cells interact via the CD44 receptor with extracellular matrix proteins such as fibronectin (FN) and osteopontin (OPN), the expression of which is increased in renal tubular epithelial cells exposed to crystals. The interactions promote crystal aggregation and growth, highlighting the pivotal role of macrophages in the pathogenesis of kidney stones [42,43].

Macrophages can differentiate into two main phenotypes with different roles in KSs, namely pro-inflammatory M1 macrophages and anti-inflammatory M2 macrophages [43,44]. M1 macrophages are typically associated with tissue damage and can enhance crystal deposition, contributing to inflammation and CaOx stone formation. In contrast, M2 macrophages have phagocytic abilities that enable them to engulf and break down CaOx crystals, thus exerting a protective effect against stone development [45,46]. The balance between these subgroups of macrophages is crucial for controlling the progression of KSs. Beyond their phenotypic differentiation, macrophages secrete various cytokines and chemokines such as interleukin-8 (IL-8), macrophage inflammatory protein-1 (MIP-1) and monocyte chemoattractant protein-1 (MCP-1), which recruit additional immune cells including, T lymphocytes, dendritic cells, neutrophils and other macrophages to the site of crystal deposition. This cascade enhances the local immune response and may facilitate further stone growth. Furthermore, macrophages release exosomes, which are small extracellular vesicles containing proteins and signaling molecules. These vesicles modulate the activation, migration and function of immune cells (especially T lymphocytes), shaping the renal inflammatory environment [47,48].

Considering the pivotal involvement of the immune system in the formation and progression of CaOx crystals, new therapeutic strategies focusing on immune modulation have gained attention. Targeting macrophage functions and their communication via exosomes could offer a promising approach to enhance crystal removal and reduce inflammation, ultimately preventing stone formation and recurrence [46]. In addition, Randall’s plaque may play an important role in reducing inflammation mediated by M1 macrophages during CaOx crystal formation [29]. Thus, maintaining a delicate balance between pro-inflammatory M1 and anti-inflammatory M2 macrophages appears crucial in preventing the inflammatory processes that promote stone development [46].

The role of immune modulation in KS prevention is an emerging area of research, although immune therapies are not currently a standard or necessary approach. The immune system contributes to stone pathogenesis by mediating inflammation and tissue injury, which can influence crystal formation [49,50]. Experimental studies have explored potential immunomodulatory interventions aiming to reduce stone formation by controlling inflammation and oxidative stress for example, mitogen-activated macrophage polarization to the M2 phenotype, use of antioxidants like curcumin, and targeting the NLRP3 inflammasome [51,52]. However, clinical evidence supporting immune therapies for the routine prevention of KSs is still lacking. Future research is essential to assess whether targeted immune interventions could become part of comprehensive KS management [53].

In this context, immunotherapy aimed at modulating the immune system could offer an effective strategy to prevent KS recurrence in selected patients. Enhancing the activity of M2 macrophages over M1 and suppressing chronic inflammation may interrupt the cascade leading to CaOx crystal nucleation and, consequently, stone formation [3]. Nevertheless, more in-depth studies are essential to fully understand these immune mechanisms and to develop effective immunotherapies for KSD.

## 4. Involvement of *E. coli* in Calcium Oxalate Nephrolithiasis: Insights into Selected Mechanisms

The presence of *E. coli* in KSD adds a new dimension to our understanding of nephrolithiasis. Studies performed indicate that IS infectious stones containing ammonium, magnesium and phosphate (struvite) are closely related to UTIs [54]. Bacteria belonging to the genera *Proteus* and *Klebsiella* contribute to the development of infectious stones due to their ability to produce urease, and thus alter urine chemistry [38]. In turn, metabolic factors and disorders play an important role in the formation of metabolic CaOx stones. This is the cause of supersaturation of mineral salts in the urine, which exceeds their solubility limit and causes crystallization of stones [55]. Uropathogenic *E. coli* (UPEC) strains, as the predominant etiologic agent of UTIs (including recurrent cases) and despite lacking the ability to produce urease (accounting for 98.6% of cases), are also involved in the formation of CaOx stones in the kidneys by stimulating renal inflammation and increasing oxidative damage in renal tubular epithelial cells [56,57,58,59]. These processes constitute a source of substrates for the heterogeneous nucleation of CaOx crystals, strengthening their adherence to renal epithelial cells and initializing crystallization [60]. Only live and intact *E. coli* bacteria promote the nucleation and aggregation of CaOx crystals. In addition, *E. coli* were detected as the most common bacterial species in both urine and CaOx KS samples in patients treated with one-stage percutaneous nephrolithotomy. For a given patient, these strains shared common genetic features, resistance genes, virulence factors, the similar sensitivity profiles to the antimicrobial agents and the same classification in phylogenetic groups [61,62]. Similar results were obtained on the basis of urine samples collected from the catheters and stone matrices in *E. coli*-infected suffering from nephrolithiasis. Bacteria were found both inside the core and nucleus of the stones. Moreover, these bacteria also exhibit adaptive properties in terms of size and protein composition to survive inside the formed stones [63]. In addition, in vivo experiments confirmed that administering *E. coli* into the urinary tract of mice leads to renal inflammation and enhances the formation of crystal deposits [36]. This emphasizes the role of *E. coli* and recurrent UTIs as etiopathogenic factors responsible for the formation of KSs [64].

Although struvite stones are classically associated with urease-producing bacteria such as *Proteus mirabilis*, recent findings reveal that a small proportion of *E. coli* strains (approximately 1.4%) may also exhibit urease activity. Nevertheless, the majority of *E. coli* strains implicated in KS formation are urease-negative and primarily associated with CaOx stones [65]. These bacteria may further aggravate urinary tract injury caused by stone movement, promoting persistent colonization and inflammation, and thereby contributing to a self-perpetuating cycle of infection and lithogenesis.

Clinical investigations have consistently identified *E. coli* in both urine and stone matrices of patients with CaOx nephrolithiasis [62,66]. In studies involving paired isolates from urine (i.e., *E. coli* urine cultures [EUC]) and stone nidus (i.e., *E. coli* stone cultures [ESC]) in the same patients, genotypic and antimicrobial susceptibility analyses revealed a high degree of concordance. These findings indicate that *E. coli* bacteria in the stones likely originate from the urinary tract and are capable of surviving and adapting to the environment inside the stone. Most strains belonged to the B2 phylogenetic group, particularly sequence types ST1193 and ST131, which are well known for their high virulence and antibiotic resistance. Proteomic profiling further showed altered expression of proteins related to stress response, carbohydrate metabolism, and DNA or protein turnover, highlighting their physiological adaptation to a more hostile and nutrient-limited niche [62]. ESC strains tended to exhibit smaller colony sizes and longer cell morphology compared to their urinary counterparts [66]. Genomic comparisons of matched EUC and ESC strains revealed striking genetic similarity, including shared virulence and resistance genes and a highly conserved genomic structure, indicating a clonal relationship. Functional assays showed that both ESC and EUC strains exhibited similar, although weaker, adhesion and invasion abilities compared to the reference UPEC CFT073 (originally isolated from the blood and urine of a patient with acute pyelonephritis) [62,67]. In addition to these phenotypic and proteomic differences, experimental data have provided a wealth of information on the molecular mechanisms by which *E. coli* can promote stone formation. Notably, persistent infection of renal tubular epithelial cells by *E. coli* has been shown to enhance the adhesion of CaOx crystals to the cell surface. This effect was mediated by the upregulation and apical translocation of ezrin, a protein that connects the membrane to the cytoskeleton. Importantly, the Rho/ROCK signaling pathway has been identified as a key regulatory axis in this process. Rho-associated protein kinase (ROCK) is involved in the regulation of ezrin in its membrane-associated form, which is essential for restructuring the apical cell membrane. Phosphorylation of ezrin at threonine residue 567 by ROCK converts the protein from an inactive to an active (phosphorylated) state, enabling its relocation to the apical membrane [68]. Pharmacological inhibition of ROCK (using the selective inhibitor Y-27632 of ezrin phosphorylation) or knockdown of ezrin expression (using a small-interfering RNA, siRNA specific for this protein) significantly reduced crystal adhesion, providing a potential mechanistic link between bacterial infection and the initiation of renal calcification [69,70].

The role of *E. coli* in KS formation is not limited to crystal adhesion. This also includes promoting oxidative damage and inflammation in kidney tissue. Studies have identified the bacterial proteins polyphosphate kinase 1 (PPK1) and flagellin as critical mediators of KS formation. These proteins act as pathogen-associated molecular patterns (PAMPs) recognized by Toll-like receptors (TLRs), particularly TLR5 in the case of flagellin, on renal epithelial cells [71]. This interaction activates the NF-κB and p38 MAPK signaling pathways and induces oxidative stress, leading to the release of proinflammatory cytokines. As a result, tissue damage is exacerbated, and a favorable environment for crystal aggregation and stone progression is created [60,72]. Other investigations have clearly shown that *E. coli* strains with flagella increase the number and size of CaOx crystals. In contrast, *E. coli* without flagella have a smaller modulatory effect on crystal formation [61,73]. This may be due to the fact that flagella allow bacteria to rotate, which is a prerequisite for increased motility (facilitating movement toward epithelial cells), bacterial adhesion and biofilm formation. In this way, *E. coli* generate an environment conducive to stone formation and provide protection against the immune response [72,74].

In turn, in vitro experiments using human renal proximal tubule (HK-2) cells showed that exposure to wild-type *E. coli* strain CFT073 (WT-CFT073) significantly increased intracellular levels of reactive oxygen species (ROS), decreased expression of superoxide dismutase 1 (SOD1) and elevated levels of 8-hydroxy-2′-deoxyguanosine (8-OHdG), indicating oxidative DNA damage. These effects were reversed in *E. coli* strains deficient in PPK1 (ΔPPK1-CFT073) or flagellin (ΔFliC-CFT073), highlighting the key role of these bacterial proteins in mediating oxidative damage. Moreover, stimulation with flagellin alone induced phosphorylation of p38 and p65 proteins and increased nuclear p65 protein expression in HK-2 cells, indicating activation of the NF-κB/p38 signaling pathway. This activation was also observed after the test cells were exposed to the WT-CT073 and was dependent on PPK1 and flagellin, as mutations in these proteins attenuated the response. Additionally, in murine models, kidney inoculation with the WT-CFT073 strain led to increased CaOx deposition, enhanced oxidative damage and upregulation of proteins associated with inflammation. These effects were reduced in ΔPPK1-CFT073 and ΔFlic-CFT073 strains, confirming the involvement of these bacterial proteins in promoting kidney stone formation through oxidative stress and inflammation [60].

These findings underscore the significant role of *E. coli* in the pathogenesis of KS, particularly through the actions of PPK1 and flagellin in inducing oxidative stress and inflammation via the NF-κB/p38 MAPK signaling pathways [60,71]. This confirms that *E. coli* bacteria occupy a unique pathogenic niche. Unlike urease-producing strains, which increase urine pH through alkalization, *E. coli* typically maintains or even contributes to an acidic urine environment, which influences its role in the pathogenesis of KSs [75]. This dual role as both a uropathogen and a stone-promoting agent places *E. coli* as a critical target for the treatment of recurrent CaOx nephrolithiasis [76].

Another study conducted by Amimanan et al. (2017) [77] also emphasized the role of *E. coli* isolated from the urine of patients with kidney stone (i.e., *E. coli*-associated with urinary kidney stones, [EUK]) in promoting CaOx crystal formation. The researchers compared EUK bacterial strains with strains originating from patients who had UTIs but did not develop stones (i.e., *E. coli*-associated urine without stones [EUU]). They found that EUK strains showed higher expression of elongation factor Tu (EF-Tu) on the surface of outer membrane vesicles (OMVs) that significantly enhanced CaOx crystal nucleation and growth, aggregation, and adhesion (by acting as structural scaffolds that interact with crystal surfaces) compared to patients with EUU. These vesicles possess a negatively charged surface that facilitates electrostatic interactions with positively charged calcium ions, thus promoting crystal aggregation. Neutralization of EF-Tu with specific monoclonal antibodies reduced these effects, highlighting its role in stone pathogenesis. Additionally, immunofluorescence staining revealed that EF-Tu was more abundantly expressed on the surface of EUK cells in contrast to EUU strains [77]. This underscores the involvement of EF-Tu in modulating CaOx crystal formation and development. Moreover, the findings indicate that certain *E. coli* strains may play an active role in KS formation and represent potential targets for therapeutic intervention.

A particularly noteworthy mechanism by which *E. coli* affects CaOx stone formation involves the activity of citrate lyase [71,78]. Citrate, a naturally occurring compound in urine, plays an important protective role by binding to calcium ions and thereby reducing their availability for crystal nucleation and growth. In addition, citrate helps alkalize urine, further inhibiting crystallization [79]. *E. coli* bypasses this host defense mechanism by producing citrate lyase, an enzyme capable of breaking down citrate into oxaloacetate and acetate. This enzymatic degradation reduces the level of citrate in the urine, tipping the balance toward CaOx supersaturation and enhancing the conditions conducive to stone formation. The reduction in citrate levels changes the urine chemical profile in favor of a more lithogenic environment, making it easier for crystals facilitating crystal formation and persistence [80]. What makes this enzymatic function particularly concerning is its potential to diminish the effectiveness of standard citrate supplementation therapies used to prevent stone recurrence [81]. By metabolizing both endogenous and exogenous citrate, *E. coli* may not only contribute to the initiation and growth of CaOx crystals but also impair a key clinical strategy aimed at mitigating stone recurrence. This interaction suggests the need to reevaluate therapeutic strategies in patients with recurrent CaOx nephrolithiasis in the presence of *E. coli* colonization (Table 1) [71].

**Figure 2:**
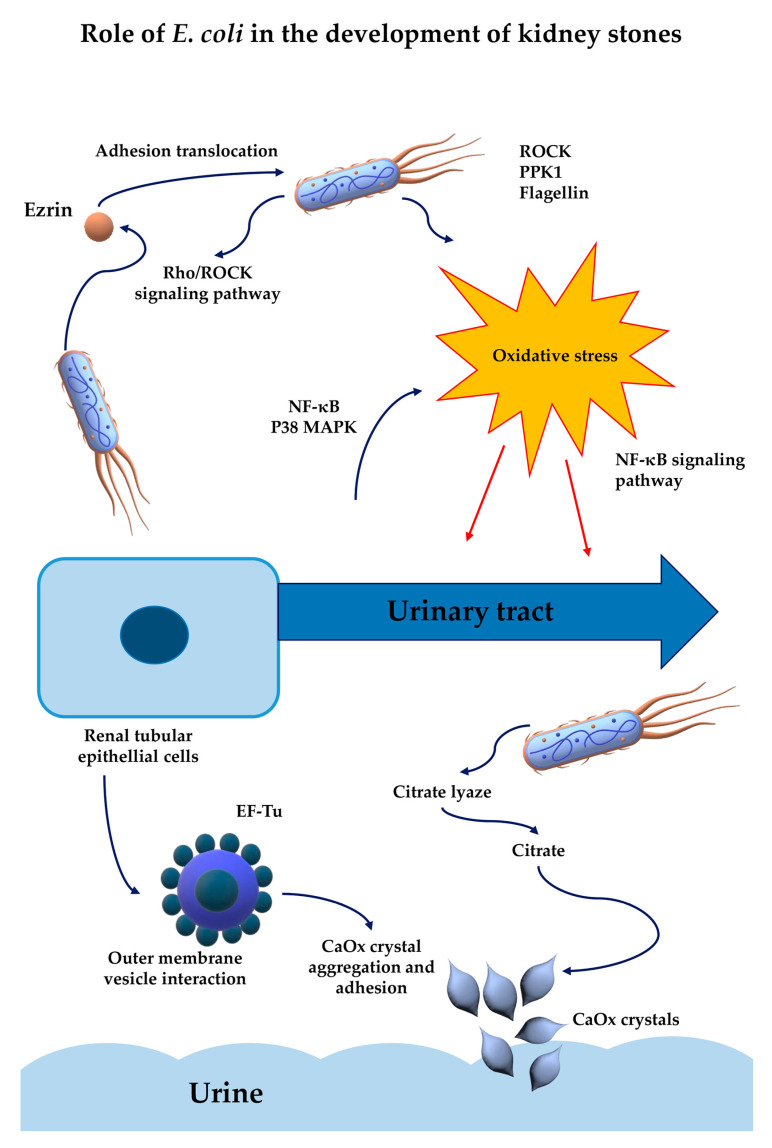
Impact of *E. coli* on the mechanisms underlying kidney stone formation [38,60,68,69,70,72,74,77,80].

Generally, these studies demonstrate the ability of *E. coli* to promote CaOx crystal growth and aggregation, though the precise mechanisms behind this effect remain poorly understood and require further in-depth research (Figure 2). In the future, integrated approaches that address both the microbial and metabolic dimensions of stone disease are warranted. Therapeutic strategies may include not only conventional antibiotic regimens but also targeted interventions aimed at disrupting bacterial adhesion, signaling pathways, or the host inflammatory environment. The recognition of the role of *E. coli* in the pathogenesis of stone disease represents a key advance in the field and encourages further research into the microbial involvement in an apparently metabolic disease.

## 5. Potential Strategies to Prevent the Development of Kidney Stones

Prevention of nephrolithiasis, particularly CaOx stone disease, is an emerging area of research that increasingly emphasizes biological and microbiological strategies. Traditional recommendations such as increasing fluid intake, reducing dietary oxalate, or prescribing thiazide diuretics offer limited efficacy and often do not prevent recurrence, which remains a major clinical concern [14]. This has prompted a shift toward more targeted approaches that modulate oxalate metabolism at its source within the gastrointestinal tract and microbiome.

In 2020, Gupta et al. [82] highlighted the therapeutic promise of bacterial enzymes, particularly those capable of degrading oxalate, a metabolic end-product that is key to CaOx stone formation. Unlike the human host, certain gut microbes possess the enzymatic machinery to metabolize oxalate, thus reducing the pool available for urinary excretion. *O. formigenes*, a strictly anaerobic bacterium, utilizes oxalates as a primary carbon source, breaking them down enzymatically in the gut before they are absorbed into the systemic circulation [82,83]. Gupta’s work outlined how the use of such bacteria or their enzymes as biotherapeutic agents could significantly reduce oxalate burden, and thus serve as a primary preventive measure against nephrolithiasis [82].

This microbial perspective was further enriched by Suman et al. (2021) [84], who emphasized the role of lactic acid bacteria such as *Lactobacillus* and *Bifidobacterium*. These types of probiotics, widely used in the food and supplement industries, have demonstrated strain-specific abilities to degrade oxalate through key enzymatic pathways including oxalyl-CoA decarboxylase (oxc) and formyl-CoA transferase (frc) [84,85]. Unlike *O. formigenes*, which is difficult to culture and maintain in the gut, these lactic acid bacteria offer higher viability and safety profiles, making them suitable for long-term preventive use. It is worth noting that some strains have shown oxalate degradation rates of up to 100% in controlled experiments [84].Their inclusion in probiotic formulations could provide an accessible, food-based approach to reduce oxalate in urine and lower the risk of stone recurrence

Furthermore, Mani et al. (2025) [86] examined the broader role of the microbiome in the pathophysiology of KS. Considerable attention has been given to how dysbiosis, a microbial imbalance, can affect oxalate metabolism and stone development. Several commensal bacteria, including *Eubacterium lentum*, *Enterococcus faecalis*, and some strains of *E. coli*, were also distinguished for their potential in oxalate degradation. Researchers noted that future therapies could involve tailored microbial consortia aimed at restoring homeostasis of the gut-urinary axis. Such targeted manipulation of microbial communities may reduce intestinal oxalate absorption and improve systemic oxalate regulation, thereby offering an integrative approach to prevention [86].

Moreover, Taheri et al. (2024) [76] synthesized the results of studies on both probiotics and herbal medicine. They found convincing evidence that oral administration of probiotic strains of *Oxalobacter*, *Lactobacillus*, and *Bifidobacterium* can lower urinary oxalate levels and mitigate crystallization processes that initiate stone formation. Additionally, various herbal extracts (*Bergenia ciliata*, *Pyrrosia lingua*, *Urtica dioica*, *Tribulus terrestris*, *Acorus calamus*) administered in animal models of nephrolithiasis have been shown to reduce kidney inflammation, protect against oxidative stress and reduce the deposition of CaOx crystals. Integrating microbiota-modulating probiotics with plant-derived anti-inflammatory agents appears to represent a synergistic and promising strategy for individuals with recurrent or high-risk profiles [76].

In addition, emerging evidence underscores the key role of intestinal bacteria that produce short-chain fatty acids (SCFAs) in modulating oxalate transport, thereby influencing the risk of KS formation. Among them, *Faecalibacterium prausnitzii* and *Roseburia hominis* have attracted interest due to their indirect but significant effects on CaOx homeostasis [87,88]. Although these species do not enzymatically degrade oxalate, their metabolic byproducts particularly butyrate alter the expression of epithelial transporters by reducing SLC26A3 expression (involved in oxalate absorption) and increasing SLC26A6 expression (which promotes oxalate secretion). This bidirectional regulation reduces net oxalate absorption and decreases urinary oxalate excretion, a key factor in preventing CaOx crystallization. Microbiome analyses consistently demonstrate reduced abundance of these taxa in stone formers, and experimental studies confirm that SCFAs supplementation reduces renal crystal deposition. Despite their therapeutic potential, clinical use of *F. prausnitzii* and *R. hominis* remains limited by the challenges of cultivation, formulation, and stable colonization [89]. Nevertheless, encapsulation technologies and microbiome engineering strategies offer promising opportunities to harness their protective effects in preventing KS.

Going forward, the application of synthetic biology offers opportunities for profound innovation and change. Wan et al. (2024) [26] provided an overview of recent advances in engineering probiotics with enhanced oxalate degradation ability. By inserting genes such as *oxdC*, *oxC*, and *frc* into microbial hosts, the researchers have developed recombinant strains that can survive harsh intestinal conditions while expressing key oxalate-degrading enzymes. For instance, engineered *Lactobacillus plantarum* expressing *oxdC* showed significantly higher oxalate degradation in vitro and in vivo. More advanced constructs include strains equipped with transporter proteins (such as OxlT) that enhance intracellular uptake and processing of oxalate. These engineered microorganisms offer precise tools for correcting oxalate imbalance and represent a step forward in prophylactic therapy for KSD [26].

## 6. Approaches to Infectious Urolithiasis: Surgical Interventions, Drug Therapies and Future Directions

Effective and individualized treatment strategies are essential to manage the complexity of infectious urinary stones and remain a key goal despite the increasing attention to prevention, especially among patients with recurrent stones. Infectious stones, particularly when associated with bacterial colonization by common pathogens such as *P. mirabilis* and *E. coli*, require an individualized and comprehensive treatment approach [71]. Surgical interventions, including extracorporeal shock wave lithotripsy (ESWL), flexible urethroscopy (FURS), and percutaneous nephrolithotomy (PCNL), are widely used, although each has its own limitations. ESWL is generally avoided in infectious cases because of the risk of spreading pathogens, while PCNL, effective for larger stones, carries an increased risk of postoperative infection, especially in patients colonized with *E. coli*. Therefore, precise selection of preoperative antibiotics based on urine cultures is crucial. Increasing antimicrobial resistance among uropathogens requires the judicious use of antibiotics and the development of new drugs, with imipenem and amikacin remaining among the more effective options [90,91].

In addition to surgical treatment, pharmacological interventions target the biochemical environment that promotes stone formation. Urease inhibitors such as acetohydroxamic acid (AHA), have shown utility but are limited by adverse effects. New compounds, including hydroxamic acid derivatives, sulfonamides, and heterocyclic agents, are being studied for their improved efficacy and safety profiles [92,93]. Adjunctive therapies such as citrate, tartronic acid, and succinate help modulate urine chemistry, while protocatechuic acid interferes with biofilm formation by *P. mirabilis*. Moreover, recent metabolomic analyses suggest the potential of using specific metabolites as therapeutic targets [94]. Ultimately, the integration of antimicrobial stewardship with metabolic modulation and advanced pharmacological strategies may provide the most effective route to treating infectious nephrolithiasis and preventing recurrence.

In conclusion, an integrated understanding of the pathogenesis and treatment of KSD, from microbial modulation to surgical and pharmacological treatment represents a significant progress in both prevention and therapy [71]. Preventive strategies focused on microbiome engineering, probiotics, and dietary modification target the earliest stages of oxalate management, offering promising non-invasive options. Meanwhile, therapeutic approaches combining targeted antibiotic use, surgical precision and metabolic intervention remain essential for patients with infectious or complex stones. Further research into novel urease inhibitors, metabolic regulators and therapeutic metabolites will be essential to improve outcomes. Together, these strategies provide a comprehensive framework for reducing the incidence, severity and recurrence of KS.

## 7. Overview of KSD Pathogenesis and Management

KSD is a urological disorder characterized by the accumulation of minerals and acid salts that crystallize and aggregate in concentrated urine. The pathogenesis of CaOx nephrolithiasis encompasses complex interactions between microbial pathogens, host epithelial cells and the immune system [3,60]. Many factors contribute to the formation of KS. Chronic conditions such as diabetes, dyslipidemia and high blood pressure are associated with an increased risk of developing this urinary disorder [4].

Emerging evidence also implicates UPEC strains as a key contributor to KS formation, extending their pathogenic role beyond UTIs to active participation in lithogenesis. Recent studies have identified *E. coli* as the most frequently detected species in both urine and KSs of patients with CaOx urolithiasis [60]. Notably, the promotion of CaOx crystal nucleation and aggregation has been observed exclusively in the presence of live and intact *E. coli* cells [61]. Consistently, matched *E. coli* isolates from urine and KSs of the same patient displayed nearly identical genotypic and phenotypic profiles, including antimicrobial susceptibility, phylogenetic classification, and the presence of virulence and resistance genes [62].

In turn, mechanistic insights revealed that colonization of renal tubular epithelial cells by UPEC activates the ROCK pathway, resulting in ezrin phosphorylation and cytoskeletal changes which enhance epithelial adhesion and promote CaOx crystal retention [68]. Inhibition of this pathway reduces crystal binding, highlighting its therapeutic potential. Additionally, EF-Tu on the surface of OMVs facilitates CaOx nucleation and growth [77], while bacterial citrate lyase depletes urinary citrate, disrupting homeostasis and promoting lithogenesis [71,78].

KSs associated with *E. coli* present a significant clinical challenge and require well-coordinated, individualized treatment strategies [71]. The growing problem of antimicrobial resistance requires careful antibiotic selection based on microbiological data, along with continued efforts to develop novel therapeutic agents [90,91]. Surgical procedures remain fundamental in clinical practice, although their use must be carefully balanced against the risk of infection and procedural complications [71]. In turn, pharmacological interventions, including urease inhibitors and newly investigated bioactive compounds, aim to disrupt stone-promoting biochemical processes while minimizing adverse effects [92,93]. Additional therapies that modulate urine chemistry and inhibit biofilm formation, such as citrate, tartronic acid, and protocatechuic acid, further support treatment efforts [94]. Preventive strategies focused on modulation of the urinary microbiome, dietary adjustments, and the use of probiotics also show potential in reducing recurrence and improving long-term outcomes [71]. These combined approaches support a more integrated and effective framework for the management of infection-associated nephrolithiasis. While current therapeutic and preventive strategies offer promising results, further research is essential to optimize treatment efficacy, develop targeted interventions, and enhance long-term prevention of infection-related KSD.

## 8. Discussion

UPEC-induced nephrolithiasis involves a multifaceted pathophysiology integrating mechanisms of bacterial adhesion, enzymatic modulation of urinary inhibitors and an abnormal immune response. Targeting these interrelated pathways through antimicrobials, immune modulators and microbiome-targeted therapies may herald a paradigm shift in the prevention and treatment of CaOx kidney stones [3,71]. Although growing evidence supports the role of UPEC in lithogenesis, significant gaps remain in our understanding of the exact molecular pathways involved. These gaps limit our ability to fully characterize the pathogenic potential of UPEC and its contribution to stone development. Nevertheless, further research is needed to define the critical factors and mechanisms by which UPEC contributes to CaOx stone formation, given its consistent presence in both urine and kidney stones.

## Figures and Tables

**Table 1:** Overview of microbial and molecular factors involved in the pathogenesis of urolithiasis [38,47,60,68,69,70,71,72,74,76,77,78,80].

Microbial and Molecular Mechanisms in Urolithiasis Pathogenesis		
Factor/Mechanism	Mode of Action	Lithogenic Impact
Urease activity (rare in *E. coli*)	Catalyzes urea hydrolysis into NH_3_ and CO_2_, increases local pH	Promotes struvite stone formation via increased Mg^2+^/NH_4_^+^/PO_4_^3−^ precipitation
Flagellar motility and adhesion	Bidirectional flagellar rotation enhances chemotaxis and adherence to epithelium and stone surfaces	Facilitates colonization, biofilm formation, and CaOx stone nucleation
PPK1/flagellin signaling	Polyphosphate kinase 1 regulates flagellin expression, induces ROS, and activates NF-κB/p38 MAPK pathways	Promotes oxidative stress, inflammation, and CaOx aggregation in renal tubules
EF-Tu on OMVs	Surface-exposed EF-Tu interacts electrostatically with CaOx crystals	Enhances nucleation, growth kinetics, and crystal aggregation
Ezrin (cytoskeletal linker)	Rho/ROCK pathway activation leads ezrin to apical membrane, binds CaOx via F-actin interactions	Stabilizes crystal adhesion and retention on tubular epithelium
Citrate lyase, enzymatic degradation of citrate	Enzymatic conversion of citrate to oxaloacetate and acetate	Reduces urinary citrate, impairing calcium chelation, promotes CaOx supersaturation
Surface morphology of CaOx stones	Rough, heterogeneous surfaces increase surface energy and bacterial/biofilm adhesion sites	Protects bacteria within stones, supporting persistent infection
Oxidative damage of renal epithelium	ROS-induced lipid peroxidation exposes phosphatidylserine and nucleation sites	Initiates heterogenous CaOx nucleation and epithelial injury
Bacterial secreted metabolites	Modulate urine supersaturation, promote aggregation, alter physicochemical environment	Foster stone matrix development and bacterial incorporation
Calcium-containing *Calcifying nanoparticles*	Mineral–protein nanoparticles that bind calcium and promote carbonate apatite biomineralization at physiological pH; induce oxidative stress and apoptosis pathways	Act as nucleation foci facilitating stone calcification; contribute to stone growth via oxidative and apoptotic mechanisms
Urinary pH modulation	Acidic pH (4.5–5.5) favors CaOx monohydrate formation and adhesion; alkaline pH supports CaOx dihydrate formation	Alters crystal type, solubility, and adhesive potential
Gut microbiota oxalate metabolism (*Oxalobacter*, *Bifidobacterium*, *Lactobacillus*)	Enzymatic degradation of dietary and endogenous oxalate	Reduces urinary oxalate load, lowering CaOx supersaturation and stone risk

## Data Availability

Not applicable.

## Abbreviations

The following abbreviations are used in this manuscript:

UTIs	Urinary tract infections
KSs	Kidney stones
KSD	Kidney stone disease
CaOx	Calcium oxalate
EQUC	quantitative urine culture
CaP	Calcium phosphate
UPEC	Uropathogenic *E. coli*
IL-8	Interleukin-8
MIP-1	Macrophage inflammatory protein-1
MCP-1	Monocyte chemoattractant protein-1
EUC	*E. coli* urine cultures
ESC	*E. coli* stone cultures
ROCK	Rho-associated protein kinase
siRNA	Small-interfering RNA
PPK1	Polyphosphate kinase 1
PAMPs	Pathogen-associated molecular patterns
TLRs	Toll-like receptors
HK-2	Human renal proximal tubule cells
WT-CFT073	Wild-type *E. coli* strain CFT073
ROS	Reactive oxygen species
SOD1	Superoxide dismutase 1
8-OHdG	8-hydroxy-2′-deoxyguanosine
EUK	*E. coli*-associated with urinary kidney stones
EUU	*E. coli*-associated urine without stones
EF-Tu	Elongation factor Tu
OMVs	Outer membrane vesicles
oxc	Oxalyl-CoA decarboxylase
frc	Formyl-CoA transferase
SCFAs	Short-chain fatty acids
ESWL	Extracorporeal shock wave lithotripsy
FURS	Flexible urethroscopy
PCNL	Percutaneous nephrolithotomy
AHA	Acetohydroxamic acid
